# Immune signatures of the POLE mutation in endometrial carcinomas: a systematic study based on TCGA data and clinical cohort validation

**DOI:** 10.3389/fonc.2023.1250558

**Published:** 2023-11-02

**Authors:** Tieyan Wang, Dan Yu, Juanjuan Wang, Ningning Zhu, Xian-bin Tang, Xiuwen Chen, Xiao-min Su, Yu-gang Huang

**Affiliations:** ^1^ Department of Pathology, Taihe Hospital, Hubei University of Medicine, Shiyan, China; ^2^ Department of Immunology, Nankai University School of Medicine, Tianjin, China

**Keywords:** endometrial carcinoma, POLE mutation, immune signatures, tumor immune microenvironment, immune infiltration

## Abstract

**Background:**

POLE is a critical biomarker for endometrial cancer (ECs) prognosis and therapeutic decision. However, the immune infiltration and immunotherapy-related gene expression in the tumor microenvironment (TME) of POLE-mutated ECs remain unresolved.

**Methods:**

The TCGA database was used to characterize the TME of POLE mutants, which primarily included immune cells and co-expression genes. We used immunohistochemistry (IHC) to determine immune cell abundance and PD-L1 expression in 104 EC tissues, including 11 POLE mutants and 93 wild-type.

**Results:**

The bioinformatic study found significant differences in gene expression of the chemokine family, immune-cell markers, and lysozyme in POLE mutants, along with immune response activation. In POLE-mutated ECs, the abundance of CD4+T, CD8+T, M1 macrophages, and dendritic cells increased considerably. Furthermore, POLE mutations may enhance immune cell recruitment or activation and lymphocyte homing in ECs. POLE mutants also had increased expression of immune-checkpoint suppressor genes such as PD-L1, CTLA-4, TIM-3, and others. The tumor mutation burden (TMB) was higher in ECs with POLE mutation. In the validation cohort, we discovered that POLE mutations were related to the immune infiltration abundance of CD8+, CD4+, and Foxp3+ cells and PD-L1 expression by IHC. The prognosis of TCGA-ECs showed that the survival time of the CD8, CD4, PD-L1, or Foxp3 over-expression subgroup of the POLE mutants was significantly prolonged compared to the down-regulation subgroup or the POLE wild-type.

**Conclusion:**

The infiltration abundance of CD8^+^ T, CD4^+^ T, Foxp3^+^ T cells, and the expression of PD-L1 harbor crucial value for the prognosis or individualized therapy of POLE-mutated ECs.

## 1 Introduction

In 2013, the molecular subtyping of endometrial carcinomas (ECs) was first proposed by The Cancer Genome Atlas (TCGA). The ECs were classified into four categories based on the genome-wide, transcriptomic and DNA methylation data, including (1) POLE hyper-mutated, (2) MSI-H (microsatellite instable-high) or mismatch repair-deficient (dMMR), (3) microsatellite stability (MSS) or low-copy numbers, (4) p53-abnormal or high-copy type (1). Investigators have further simplified it to ProMisE typing by combining clinical practice and the feasibility of pathological testing, namely POLE-mutated, MSI-H/dMMR, p53 wild-type and p53-abnormal (2–4). The catalytic subunit of DNA polymerase ϵ (POLE), repairing mistakes during DNA replication, is one of the critical molecular biomarkers in ECs (2). The structure of the POLE gene consists of 49 exons, with exons 9-14 being the central region where the major pathogenic mutations occur. More than 80% of the pathogenic variants in the POLE gene occur in exons 9 and 13, with five common hotspot mutations, including P286R, S297F, V411L, A456P, and S459F, accounting for 95.3% of the known pathogenic variants in the POLE gene (5–7).

The mutation status of POLE has considerable guiding value for ECs’ prognosis and therapy strategy selection (8). The NCCN Clinical Practice Guidelines: Uterine Neoplasms (version 1, 2023) (9) and related studies have proven that POLE-mutated ECs harbored a high tumor mutational burden (TMB), infrequent recurrence, and a favorable prognosis. The POLE-mutated ECs, considered in the low-risk group, may be addressed for follow-up after surgery to avoid adjuvant therapy (10, 11). Determining the mutation status of the POLE is practical guidance for the prognosis of ECs and the choice of therapeutic strategies (12, 13). However, studies on immune cell infiltration and expression of immunotherapy-related genes in the microenvironment of POLE-mutant EC tumors have rarely been reported. Furthermore, the current literature is highly biased toward low-staging cases, and the role of POLE gene mutations in driving patient decision-making is in its infancy. Thus, we intend to investigate the potential impact of immune cell infiltration on the prognosis of POLE-mutated ECs, provide novel insights into the molecular mechanism of POLE mutation in the development of ECs, and furnish a theoretical foundation for the precise therapeutic approach of ECs based on TCGA data and clinical specimens.

## 2 Materials and methods

### 2.1 Human EC specimens and reagents

Formalin-fixed and paraffin-embedded (PPFE) from 104 cases of ECs, including 11 POLE mutants and 93 POLE wild-type, were retrospectively collected from December 2020 to October 2022 from the archived pathology department of Taihe Hospital. Inclusion criteria: First, all specimens were obtained from patients diagnosed with ECs by pathologists, and the diagnosis was based on the 2020 WHO classification of female genital oncology according to the International Federation of Gynecology and Obstetrics (FIGO) stage. Second, all relevant patients were not treated with radiotherapy or chemotherapy before surgery.

### 2.2 Immunohistochemistry staining

The primary antibody PD-L1 (22C3) (Ready-to-use, SK006) was purchased from Agilent Dako, Denmark. The CD4 (Ready-to-use, RMA-0620) and CD8 (Ready-to-use, MAB-0021), Foxp3 (Ready-to-use, MAB-1004), CD56 (Ready-to-use, MAB-0743), and horseradish peroxidase-HRP-labeled anti-rabbit secondary antibody (Ready-to-use, KIT5230), were purchased from MXB Biotechnologies Company, Fuzhou.

The IHC assay for the immune cells, including CD4/CD8/Foxp3 T cells and CD56 NK cells, was performed using the EnVision two-step method. Paraffin tissue sections of 3 μm were taken and hot-repaired with EDTA (pH 9.0); the primary antibodies were incubated for 1 h at 37°C and secondary antibodies for 30 min at 37°C. After washing, DAB color development, hematoxylin re-staining, differentiation, dehydration, transparency, and neutral gum sealing were performed. The expression of target proteins was observed under the optical microscope. Two professional pathologists scanned the slices and scored the immunohistochemical results by the double-blind method.

The IHC score of the target protein (CD4, CD8, Foxp3 and CD56) was assessed based on the target protein’s staining intensity and range under the 100× field of view of the light microscope. Staining range: 0 (none), 1 (1%-10%), 2 (11%-50%), 3 (51%-80%) and 4 (81%-100%); staining intensity: 0 (absent), 1 (weak), 2 (moderate) and 3 (strong). The final IHC score was yielded by the multiplication of range and intensity scores, and cases with IHC score ≥4 were considered as high expression of the target protein and the rest as low expression. The clinical correlation assay was performed based on the IHC score, combined with the clinicopathological parameters of the ECs.

The IHC of PD-L1 was performed according to the reagent manufacturer’s instructions. The PD-L1 antibody (22C3) (Ready-to-use, SK006) was incubated for 0.5 h at 37°C, and then EnVision™ FLEX HRP visualization system was applied for 0.5 h incubation at room temperature. PD-L1 combined positive score (CPS) (14): PD-L1 expression was defined as partial or complete membranous staining in carcinoma cells and membranous or cytoplasmic staining in immune cells. The percentage of PD-L1 positive carcinoma and immune cells was estimated separately and combined. To calculate the CPS, we divided the total number of PDL1-positive cells (carcinoma cells, lymphocytes, and macrophages) by the number of viable carcinoma cells multiplied by 100. The cutoff for positive PD-L1 expression was set at ≥1% for all the scorings.

### 2.3 Information mining in tumor databases via bioinformatics strategy

The POLE mutation data, expression matrix and clinical information associated with 528 ECs were extracted from the TCGA database. Correlations between POLE gene mutation status and patients’ age, histological subtype, survival status, infiltration status, other pathological parameters, and patient prognosis were performed with clinicopathological information. In addition, the type and locus of POLE mutations, the correlation between mutation status and tumor mutation burden (TMB), and three different immune scoring-related algorithms (Xcell (15), TIMER (16), and CIBERSORT (17)) were analyzed using the Assistant for Clinical Bioinformatics Platform (www.aclbi.com) containing in the correlation between POLE mutation status and immune cell population abundance; the signaling pathways associated with POLE mutations with significant differential gene expression were analyzed using Funrich software (18). The TISIDB online service platform (http://cis.hku.hk/TISIDB/) was used to assess the immune cell infiltration associated with POLE mutations and cytokine expression (19).

### 2.4 Statistical analysis

SPSS 25.0 (IBM SPSS Inc., Chicago, IL) or GraphPad Prism 8.0 (San Diego, CA) were applied for statistical analysis. Independent samples t-test was used for comparison between the two groups. The χ^2^ test or Fisher’s exact test was used to compare two groups of counting data. Kaplan-Meier method and Log-rank test were used for survival analysis according to the follow-up information of patients. Univariate Cox (uni-cox) and multiple Cox (multi-cox) regression models were used to analyze the effect of POLE mutation status on survival and clinical characteristics (age, stage, etc.). *P*< 0.05 was considered a statistically significant difference (ns=non-significant, * *P*<0.05, ** *P <*0.01, *** *P <*0.001).

## 3 Results

### 3.1 Analysis of prognostic and pathological characteristics of POLE mutation status in EC tissues based on the TCGA database

**Figure 1:**
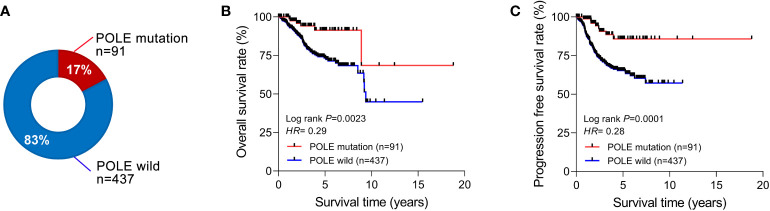
Correlation analysis of POLE mutation with clinical parameters and prognosis. **(A)** Percentage of POLE gene mutations in all TCGA-ECs. The overall survival rate **(B)** and progression-free survival rate **(C)** of ECs with POLE mutation and wild-type.

Among the 528 ECs in the TCGA database, the percentage of POLE-mutated ECs in all cases was 17% (91/528, **Figure 1A**). The Kaplan-Meier assay indicated that the overall survival rate (OS) and progression-free survival rate (PFS) of POLE-mutated ECs predicted a favorable clinical outcome, compared to the POLE wild-type (HR=0.29 and 0.28, respectively, all *P*<0.01; **Figures 1B, C**).

### 3.2 Analysis of the POLE mutation profile based on TCGA-ECs

**Figure 2:**
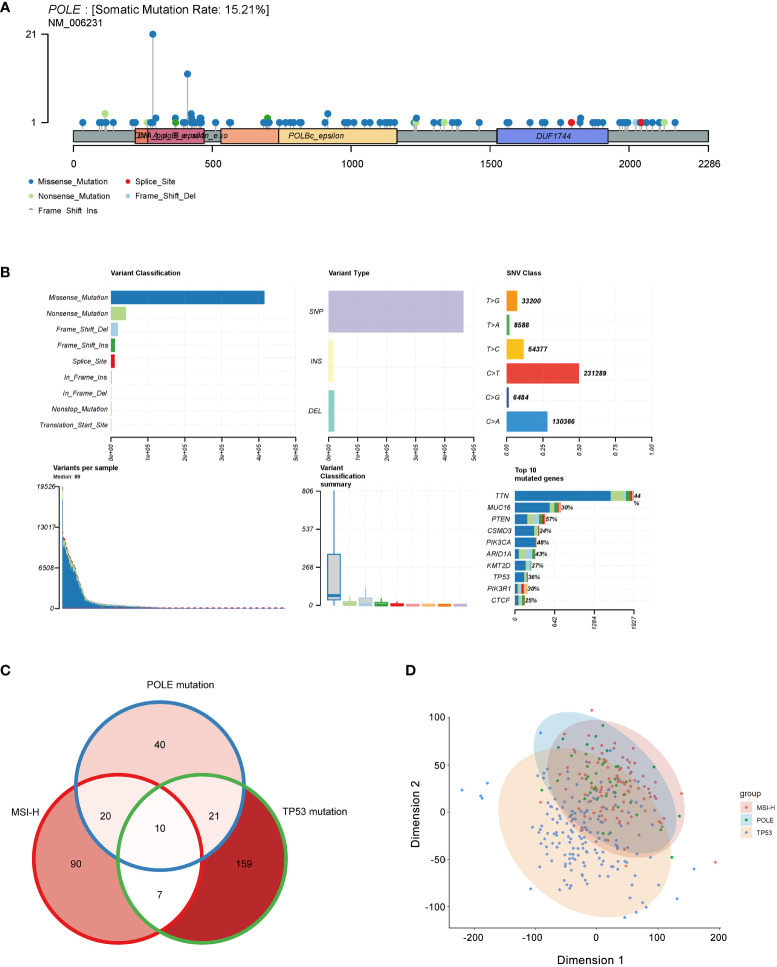
Mutation landscape of POLE mutation in ECs. **(A)** Distribution of POLE mutations in a lollipop plot. **(B)** The cluster summary graph shows the distribution of variants according to variant classification, type and SNV category. The bottom (from left to right) indicates the mutation load for each sample (variant classification type). The stacked bars show the top ten mutated genes. **(C)** Veen diagram of individual contained both TP53 mutations, MSI-H, or POLE mutations. **(D)** The principal component analysis (PCA) of patients with TP53 mutations, MSI-H, or POLE mutations.

The analysis revealed that the somatic mutation rate of POLE in TCGA-ECs was 15.21% (**Figure 2A**); we could infer that the percentage of germline POLE mutations was about 2.02%. Somatic mutations in POLE were scattered in 49 exons, and the most dominant type was missense mutations, accounting for over 80% (**Figure 2B**). Then, we investigated cases where an individual contained both TP53 mutations, MSI-H, or POLE mutations. The Veen diagram showed that only a few individuals had two or three subtypes of these mutations (**Figure 2C**). The principal component analysis (PCA) revealed significant heterogeneity of tumors with TP53 mutant subtypes compared to tumors with MSI-H/POLE mutations (**Figure 2D**).

### 3.3 Correlation analysis of POLE mutation and clinicopathological characteristics in ECs

In 528 ECs, analysis of the POLE mutants and clinicopathological signatures showed that the POLE mutation status was significantly correlated with the age, histological subtype, and survival status (alive or dead) of ECs (*P <*0.001, *P*=0.002, and *P*=0.008, respectively; **Table 1**), but not with FIGO stage and invasive status (all *P*>0.05, **Table 1**). Then, uni-cox and multi-cox regression analyses were applied to investigate the correlation between POLE mutation status and clinical characteristics such as patient age, histological subtype, and FIGO stage on the OS and PFS of EC cancer patients.

**Table 1:** Analysis of POLE mutation status and clinicopathological characteristics in TCGA-ECs (n=528).

Clinicopathological characteristics	n	POLE		Statistic	*P* value
		Wild-type (n=437)	Mutation (n=91)		
Age, median ((IQR))		64 (58, 72)	59 (53, 68.25)	23944.5	<0.001 ^a^
FIGO stage				1.069	0.78 ^b^
I	329	269	60		
II	51	43	8		
III	121	101	20		
IV	27	24	3		
Histological subtypes					0.02 ^c^
Endometrioid	395	313	82		
Serous	111	105	6		
Mixed	22	19	3		
Not available	54	45	9		
Survival status				6.96	0.008 ^b^
Survive	441	356	85		
Death	87	81	6		

^a^, Wilcoxon rank-sum test; ^b^, χ^2^ test; ^c^, Fisher test. IQR, Interquartile Range.

For the OS of all ECs (n=528), the uni-cox regression analysis indicated that POLE mutation status, age, and FIGO stage correlated with ECs’ prognosis (all *P*<0. 05, **Table 2**); the multi-cox regression analysis revealed that POLE mutation status and FIGO stage might be independent prognostic factors for all ECs (all *P*<0.01, **Table 2**). Besides, for the OS of POLE mutation ECs (n=91), the FIGO stage was not an independent risk factor(*P*>0.05, **Table 3**). For the PFS of all ECs (n=528), the uni-cox analysis suggested that POLE mutation status and FIGO stage correlated with ECs’ prognosis (all *P*<0. 01, **Table 4**); the multi-cox analysis indicated that POLE mutation status and FIGO stage might be independent prognostic factors (all *P*<0.001, **Table 4**). Interestingly, for the PFS of POLE mutation ECs (n=91), the FIGO stage might be an independent risk factor (*P*<0.05, **Table 5**).

**Table 2:** Univariate Cox (uni-cox ) and multivariate Cox (multi-cox ) regression analysis of OS in TCGA-EC (n=528).

Clinicopathological characteristics	Uni-cox		Multi-cox	
	HR (95%CI)	*P* value	HR (95%CI)	*P* value
POLE mutation status (Mutation vs wild)	0.299 (0.131-0.686)	0.004	0.296 (0.126-0.693)	0.005
Age	1.038 (1.016-1.060)	<0.001	1.020 (0.998-1.043)	0.082
Histological subtypes (Endometrioid vs others)	2.766 (1.181-6.482)	0.019	1.502 (0.626-3.601)	0.36
FIGO stage (III+IV vs I+II)	3.746 (2.451-5.725)	< 0.001	4.332 (2.806-6.689)	< 0.001

**Table 3:** Univariate Cox (uni-cox ) and multivariate Cox (multi-cox ) regression analysis of OS in POLE mutation EC (n=91).

Clinicopathological characteristics	Uni-cox		Multi-cox	
	HR (95%CI)	*P* value	HR (95%CI)	*P* value
Age	1.085 (0.994- 1.186)	0.067		
FIGO stage (III+IV vs I+II)	3.717 (0.671- 20.6)	0.133		

**Table 4:** Univariate Cox (uni-cox ) and multivariate Cox (multi-cox ) regression analysis of PFS in TCGA-EC (n=528).

Clinicopathological characteristics	Uni-cox		Multi-cox	
	HR (95%CI)	*P* value	HR (95%CI)	*P* value
POLE mutation status (Mutation vs wild)	0.317 (0.154-0.652)	0.002	0.289 (0.141-0.596)	<0.001
Age	1.006 (0.989-1.024)	0.473		
FIGO stage (III+IV vs I+II)	3.612 (2.475-5.721)	<0.001	3.773 (2.582-5.513)	<0.001

**Table 5:** Univariate Cox (uni-cox ) and multivariate Cox (multi-cox ) regression analysis of PFS in POLE mutation EC (n=91).

Clinicopathological characteristics	Uni-cox		Multi-cox	
	HR (95%CI)	*P* value	HR (95%CI)	*P* value
Age	1.034 (0.972-1.101)	0.287		
FIGO stage (III+IV vs I+II)	7.334 (1.478-36.384)	0.015	13.668 (2.487-75.121)	0.0026

### 3.4 Bioinformatics analysis of the immunomodulatory role and immune infiltration in POLE mutated ECs

**Figure 3:**
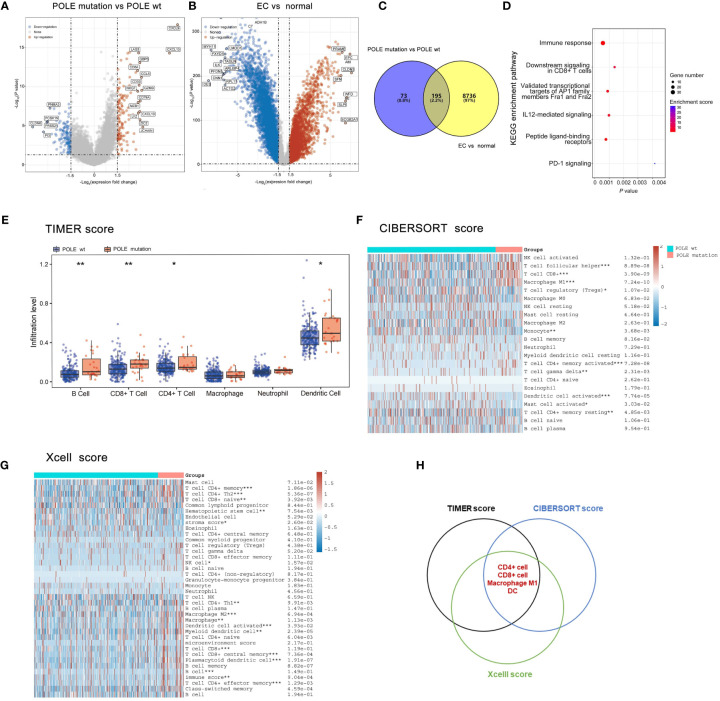
Analysis of differentially expressed genes, signaling pathways and immune cell abundance in POLE-mutated in ECs. **(A)** Screening of differentially expressed genes related to POLE mutation; **(B)**. Identification of differently expressed genes in EC compared with the adjacent samples; **(C)** Co-differently expressed genes in A and B; **(D)** Gene enrichment analysis of Kyoto Encyclopedia of Genes and Genomes (KEGG) in co-differently expressed genes. **(E–G)** The abundance of immune cell populations in POLE-mutated ECs via three different algorithms, including TIMER **(E)**, CIBERSORT **(F)**, and Xcell **(G)**; **(H)** Veen diagram of **(E–G)**. ns, non-significant, *P <0.05, **P <0.01, ***P <0.001.

The differentially expressed genes of POLE mutations vs POLE wild-type or ECs vs normal were investigate based on the analysis of POLE mutation status and transcriptome matrix in TCGA-ECs, and the threshold were set as: |gene mRNA expression difference| ≥1.5-fold and *P*>0.05. It showed that significantly highly expressed in POLE mutants included chemokine family (e.g., CXCL9, CXCL10, CXCL13, CCL5, etc.), immune cell surface markers (e.g., CD8A, CD3D, etc.) and lysozyme genes (Lysozyme, LYZ) (**Figures 3A, B**). Then, 195 co-differently expressed genes were expressed abnormally in POLE-mutated ECs (**Figure 3C**). Moreover, KEGG enrichment analysis showed that the most enriched signaling pathways in POLE mutants included immune response, CD8^+^ T cell signaling, PD-1 signaling, etc. (all *P*<0.01, **Figure 3D**). Moreover, the abundance of immune cell populations associated with POLE-mutated ECs were assessed using three different algorithms, including TIMER (**Figure 3E**), CIBERSORT (**Figure 3F**), and Xcell (**Figure 3G**). Consequently, the Veen diagram depicted that the abundance of CD4^+^ T cells, CD8^+^ T cells, M1-type macrophages, and dendritic cells (DC) was significantly higher in POLE-mutated ECs compared to POLE wild-type (all *P*<0.05, **Figure 3H**).

### 3.5 Analysis of tumor immune microenvironment and immune-checkpoint suppressor expression associated with POLE mutation

**Figure 4:**
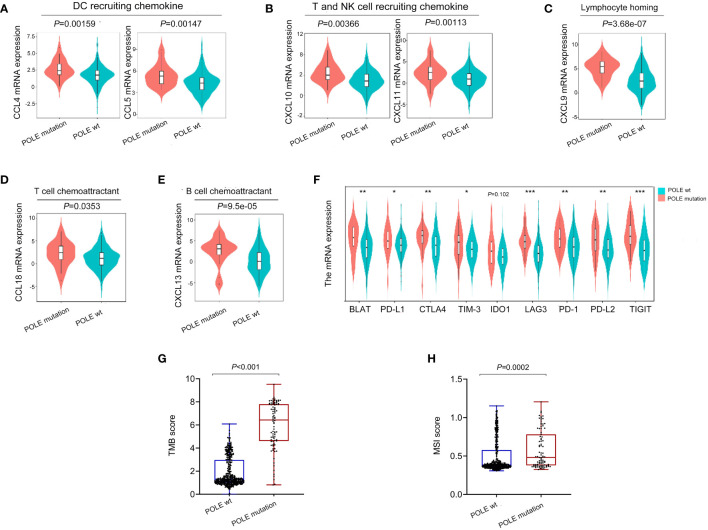
Analysis of immune cytokines and immune-checkpoint suppressor gene expression related to POLE mutation. **(A–E)** Analysis of correlation between POLE mutation and cytokines related to immune cell biological behavior (dendritic cells, NK cells, T cell recruitment, T/B cell activation, lymphocyte homing) based on the TISIDB platform; **(F)** Analysis of the expression of immune-checkpoint suppressor genes related to the POLE mutation. **(G, H)** The analysis of tumor mutation burden (TMB, **G**) and microsatellite instability (MSI, **H**) score in POLE mutants and POLE wild-type. ns, non-significant, *P <0.05, **P <0.01, ***P <0.001.

As analyzed in the TISIDB platform, a significant positive correlation was revealed between POLE mutations and cytokines related to immune cell biological behavior (dendritic cells, NK cells, T cell recruitment, T/B cell activation, lymphocyte homing) in ECs (all *P*<0.001, **Figures 4A–E**). In addition, various immune-checkpoint suppressor genes, including PD-L1, CTLA-4, TIM-3, etc., were significantly overexpressed in POLE mutants compared to POLE wild-type (all *P*<0.05, **Figure 4F**). Besides, it demonstrated that the TMB and MSI scores in POLE-mutated ECs were significantly higher than those in POLE wild-type (*P*<0.001, **Figures 4G, H**).

### 3.6 Analysis of immune cell markers associated with POLE mutants in our validation cohort

**Figure 5:**
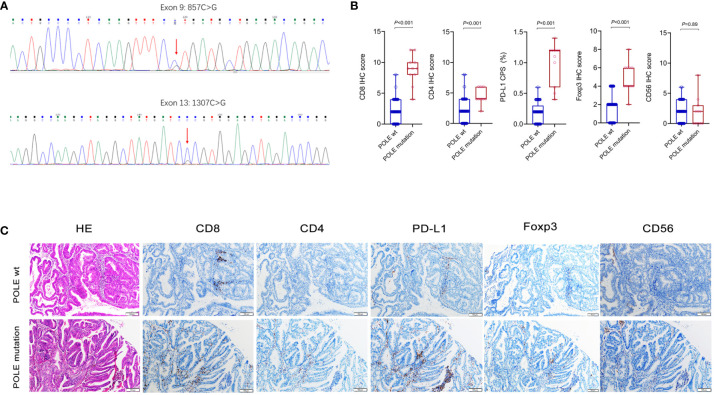
Analysis of immune cell markers associated with POLE mutants in our validation cohort. **(A)** Assay of POLE mutations by Sanger-PCR sequencing method, including exon 9 and exon 13 mutations in clinical tissues. **(B, C)** IHC assay of CD8, CD4, PD-L1, Foxp3 and CD56 in EC with POLE wild-type and mutants.

We collected 104 ECs retrospectively and designed specific primers for exons 9, 11, 13 and 14 of the human POLE gene. Consequently, we screened out 11 samples with POLE point mutations by Sanger-PCR sequencing method, mainly including 4 cases of exon 9 mutations and 7 cases of exon 13 mutations (**Figure 5A**). The percentage of POLE mutations was 10.58% (11/104) in EC samples. IHC assay of the 11 POLE mutants and 93 POLE wild-type showed that CD8^+^ T cells, CD4^+^ T cells, PD-L1^+^ carcinoma or immune cells, and Foxp3^+^ T cells had significantly higher IHC scores in POLE mutants compared to POLE wild-type (all *P*<0.05; **Figures 5B, C**), while IHC scores of CD56^+^ NK cells were not statistically differences (*P*>0.05).

In clinical samples, correlation analysis based on the POLE mutation status with IHC score and clinicopathological parameters showed that the POLE-mutated ECs were significantly correlated with high abundance of CD8, CD4, and PD-L1 (all *P* < 0.001, **Table 6**) but not with age, FIGO stage, or histological subtype (all *P* > 0.05, **Table 6**).

**Table 6:** Analysis of POLE mutation status and clinicopathological characteristics in our validation cohort (n=104).

Clinicopathological characteristics	POLE		Statistic	*P* value
	Mutation (n=11)	Wild-type (n=93)		
Age, mean ± SD	61.909 ± 14.862	65.978 ± 12.572	0.99	0.32^a^
BMI, mean ± SD	30.139 ± 8.4996	34.342 ± 8.2795	1.58	0.012^a^
FIGO stage			1.35	0.51^b^
I	2	11		
II	4	23		
III	5	59		
Histological type			3.89	0.143^b^
Endometrioid	11	68		
Serous	0	21		
Mixed	0	4		
CD8 IHC score, median	9 (8, 10)	2 (0, 4)		0.572^c^
CD4 IHC score, median	4 (4, 6)	2 (0, 4)		< 0.001^c^
PD-L1 IHC score, median	8 (5, 9)	2 (0, 2)		< 0.001^c^
Foxp3 IHC score, median	6 (4, 8)	2 (0, 2)		< 0.001^c^
CD56 IHC score, median	2 (0, 2.5)	2 (0, 4)		< 0.001^c^

^a^, Student-t test; ^b^, χ2 test; ^c^, Wilcoxon rank-sum test; SD, standard deviation. BMI, body mass index.

### 3.7 Influence of CD8^+^, CD4^+^ PD-L1^+^, and Foxp3^+^ expression abundance on the prognosis of POLE wild-type or POLE-mutated ECs

**Figure 6:**
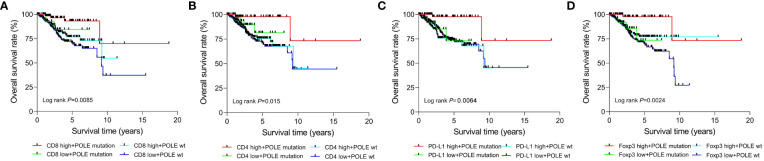
Survival analysis of CD8 **(A)**, CD4 **(B)**, PD-L1 **(C)**, and Foxp3 **(D)** high-expression or low-expression subgroups in ECs with POLE mutation or POLE wild-type.

According to the median expression, the TCGA-ECs were divided into high-expression and low-expression subgroups of CD8, CD4, PD-L1 or Foxp3, respectively. Prognostic analysis of the CD8 subgroup showed that the CD8 high expression and POLE mutant subgroup had significantly prolonged survival, and the CD8 low expression and POLE wild type subgroup suffered the shortest survival in ECs (*P*=0.0085, **Figure 6A**). For the CD4 subgroup, the CD4 high expression and POLE mutant subgroup had the significantly most prolonged survival and the CD4 low expression and POLE wild-type subgroup suffered the shortest survival (*P*=0.015, **Figure 6B**). For the PD-L1 subgroup, the PD-L1 high expression and POLE mutant subgroup had the significantly most prolonged survival, and the PD-L1 low expression and POLE wild-type subgroup suffered the shortest survival (*P*=0.0064, **Figure 6C**). For the Foxp3 subgroup, the Foxp3 high expression and POLE mutant subgroup had the significantly most prolonged survival; The Foxp3 low expression and POLE wild type subgroup suffered the shortest survival time (*P*=0.0024, **Figure 6D**).

## 4 Discussion

Endometrial cancer (ECs) is the most prevalent gynecological malignancy in affluent countries (8, 20). In 2020, ECs added 82,000 cases and 16,600 deaths, and the incidence is increasing annually in China (21, 22). Studies have reported that approximately 80% of ECs were diagnosed in the early stages without metastasis, and the 5-year survival rate is over 95% (23). For patients with local spread or distant metastases, the 5-year survival rate was 68% or 17%, respectively (24). Thus, early diagnosis and treatment can significantly enhance the prognosis survival of ECs (25). POLE serves a significant role in repairing DNA replication mistakes, and POLE mutation is one of the essential biomarkers currently recommended for treating ECs (6, 8, 26). Based on the TCGA database, the bioinformatics analysis confirmed a significantly improved prognosis for POLE-mutated ECs. It is consistent with prior research findings (27–29). We revealed that the differentially expressed genes of POLE mutants were primarily enriched in the immune response pathway. The pathogenic POLE mutation resulted in mistakes in DNA replication that were difficult to repair, resulting in an accumulation of DNA mismatches that eventually caused a high number of gene expression anomalies and carcinogenesis. The immune system of the host will recognize the accumulated errors and unique antigens created by cancer cells, resulting in the activation, recruitment, and infiltration of immune cells. Nevertheless, tumors need to construct an immunosuppressive microenvironment to fight against the immune system’s surveillance, thus achieving immune escape. Thus, these immune infiltrating cells and immune**-**checkpoint suppressor genes are potential therapeutic targets for ECs. However, the immune infiltrating cell abundance and immune**-**checkpoint suppressor gene expression in ECs are not clearly defined.

The TCGA assay revealed significant DEGs and immune response activation in POLE mutants. Specifically, the abundance of CD4^+^ T, CD8^+^ T, M1 macrophages and DCs was significantly upregulated in POLE-mutated ECs. In POLE mutants, various immune checkpoint suppressor genes, including PD-L1, CTLA-4, TIM-3, etc., were overexpressed. The clinical validation cohort confirmed the above findings, with upregulated CD8^+^, CD4^+^, and Foxp3^+^ cells and PD-L1 expression in the POLE mutants ECs. The prognosis of TCGA-ECs showed that the survival time of CD8, CD4, PD-L1 or Foxp3 over-expression subgroup of the POLE mutants was significantly prolonged, compared with the down-regulation subgroup or the POLE wild-type. It further hinted that CD8^+^T, CD4^+^T, Foxp3^+^ T cell infiltration abundance and overexpressed PD-L1 might have potential prognostic or therapeutic value for POLE-mutated ECs (30). Therefore, increasing the abundance and infiltration of immune cells (especially CD8^+^ T and CD4^+^ T cells) and blocking the PD-L1 expression will probably be a promising strategy to offer precise treatment for POLE-mutated ECs. Besides, tumor mutation burden (TMB) has aroused attention in immunotherapy as a biomarker for predicting response to PD-1 antibody therapy (31). The NCCN Clinical Practice Guidelines of ECs suggested that the preferred second-line biomarker-directed systemic treatment options include Levatinib/Palmtuzumab (level 1 evidence, recommended for advanced or relapsed patients who have progressed on prior systemic therapy, are unable to undergo radical surgery or radiotherapy, and are not in MSI-H or dMMR), and Palmtuzumab (tumors with TMB-H or MSI-H/dMMR). Other recommended agents include Navumab (tumors with dMMR/MSI-H), dostarlimab-gxly (tumors with dMMR/MSI-H), and Larotrectinib or Entrectinib tumors with the NTRK gene fusion) (level 2B evidence), Avelumab (tumors with dMMR/MSI-H) and Cabozantinib (multi-targeted small molecule tyrosine kinase inhibitor) (9).

The clinical development of checkpoint inhibitor-based immunotherapies has ushered in an exciting age of anticancer therapy. Immunotherapeutic markers widely recognized in clinical studies mainly included PD1/PD-L1 expression, tumor-infiltrating lymphocytes, tumor mutation burden, and immunogenetic features (32, 33). Our study presented that TMB and MSI scores were significantly higher in POLE mutant samples, suggesting that POLE mutated ECs with infiltrating immune cells, especially CD4^+^, CD8^+^, and Foxp3^+^ T cells, may have a more positive response to immune-checkpoint inhibitors. However, not all POLE mutations respond significantly to immunosuppression, and the underlying reasons remain unsolved. As previous reports, abundant Treg cell infiltration into tumors is associated with poor clinical outcomes in various types of cancers, including colorectal cancers (34), breast cancer (35), etc. Surprisingly, the role of Treg cells is controversial in ECs, in which Foxp^3+^ T cell infiltration indicated a better prognosis. Iwasaki T et al. demonstrated that high infiltration of cells with low-intensity Foxp3 expression in the invasive front is a favorable prognostic factor in Merkel cell carcinoma (36). Furthermore, a functionally distinct subpopulation of tumor-infiltrating Foxp3^+^ T cells, secreting interleukin (IL)-12 and transforming growth factor (TGF)-β, showed significantly favorable prognosis in colorectal cancers (37). This contradiction suggested that Foxp3^+^ T cell subsets play a complex and critical role in the development of ECs, implying that this class of Foxp3^+^ T cell subsets could be a promising prospective therapeutic target for ECs. Further, POLE mutations directly sensitized tumors to immune checkpoint blockade therapy (ICB) response. Ma X et al. demonstrated that in mice model, POLE/POLD1 mutation-associated alterations promoted the production of T cell receptor (TCR) contact residues with increased hydrophobicity by tumor cells, which might facilitate T cell recognition of tumor cells (38). Thus, integrating multiple therapeutic modalities based on the characteristics of the tumor immune microenvironment would be an effective avenue for future antitumor therapy.

Generally, the impact of POLE mutation on the whole EC cells and tumor microenvironment is intricate and complex, including not only the activation or infiltration of CD8^+^ T and CD4^+^ T cells or the abnormal expression of PD-L1 and other genes but also the alteration of gene expression patterns or signaling pathways. Furthermore, the prognosis of POLE-mutated ECs with different individuals and disease foci is inconsistent. Thus, clarifying the expression of the immune infiltration and immunotherapy-related genes is imperative to achieve precise therapeutic strategies for ECs. Otherwise, the exact cause of the triggering mutations in the POLE gene is unknown. Finally, the current study has certain limitations: Firstly, the clinical sample size of POLE-mutated ECs is relatively small, and the mutation classifications are relatively single. Thus, an enlarged sample size and the Next Generation Sequencing (NGS) method are essential to discover new mutation sites. Then, the prognostic assessment in this essay is mainly based on the bioinformatics strategy of the TCGA database, and its exact prognostic value has to be further confirmed by clinical practice. Moreover, for the existing studies and the samples in this study, most of the POLE mutations occurred in the early stage (mainly FIGO G1/G2) (39), while the studies related to advanced-stage patients were scarce. Therefore, further in-depth studies on advanced-stage patients, including those with POLE mutations, were warranted.

For ECs with early-stage (FIGO G1/G2) POLE mutant, conservation may be considered, or surgical resection as the main treatment modality, and adjuvant treatment (radiotherapy and chemotherapy) was not recommended unless necessary. In contrast, for early-stage POLE wild-type ECs, adjuvant therapy was required after surgery. Thus, for early-stage patients, the practical value of POLE mutations is to assess prognosis and direct treatment (40). In this paper, we analyzed the POLE-associated immune cell infiltration and the characteristics of immune activity. We intend to explore their potential value for mitigating disease progression or recurrence in ECs. The immune-related genes in this study may be potential targets for inhibiting ECs recurrence or progression. For advanced ECs with POLE mutations, numerous studies have advocated combination therapy, including immunotherapy, to reduce the adverse effects of chemotherapy (41, 42). It is also the viewpoint presented in the current study. Finally, the prognostic value of various POLE mutant sites or categories, as well as their clinical significance for prognostic or targeted biomarkers, remain to be studied further. As a result, more researchers and multi-omics investigations are needed to uncover the hidden mysteries.

## 5 Conclusion

Based on the TCGA database, we investigated the tumor microenvironment (TME) of POLE mutations, focusing on immune cells and co-expression genes. Then, in our validation cohort, we investigate for immune cell abundance and PD-L1 expression in ECs via immunohistochemistry (IHC). It was concluded that POLE-mutated ECs presented a positive prognosis and are closely related to immune cell infiltration. POLE-mutated ECs were mostly implicated in the immune response and the PD-1 signaling pathway. Upregulation of immune cell infiltration and PD-L1 expression in POLE-mutated ECs may have prognostic or therapeutic implications. As a result, increasing the number and infiltration of immune cells (particularly CD8+ T and CD4+ T cells) and inhibiting the expression of genes like PD-L1 would most likely be an attractive strategy for treating POLE-mutated ECs.

## Data availability statement

Publicly available datasets were analyzed in this study. This data can be found here: All publicly available datasets in this work are available from TCGA (https://portal.gdc.cancer.gov/); further inquiries can be directed to the corresponding authors.

## Ethics statement

All clinical specimens were collected by informed consent (IFC) from patients or family members, and this study has been supported and approved by the Ethics Committee of Taihe Hospital. The studies were conducted in accordance with the local legislation and institutional requirements. The participants provided their written informed consent to participate in this study.

## Author contributions

TW, Y-GH, and DY designed the study and wrote the draft. JW and NZ performed the IHC assay and statistical analysis; Y-GH and X-MS supervised this study, participated in the study design, and revised the manuscript. X-BT furnished helpful advice on methods and chart preparation. XC assisted in revising the manuscript and provided many important suggestions. All authors have read and approved the final version of the manuscript. All authors contributed to the article and approved the submitted version.
